# Individual, household, and community level barriers to ART adherence among women in rural Eswatini

**DOI:** 10.1371/journal.pone.0231952

**Published:** 2020-04-28

**Authors:** Nozipho Becker, Lorraine S. Cordeiro, Krishna C. Poudel, Thokozile E. Sibiya, Aline G. Sayer, Lindiwe N. Sibeko

**Affiliations:** 1 Department of Nutrition, University of Massachusetts, Amherst, Massachusetts, United States of America; 2 Department of Food and Nutrition Sciences, University of Eswatini, Luyengo, Kingdom of Eswatini; 3 Department of Health Promotion and Policy, University of Massachusetts, Amherst, Massachusetts, United States of America; 4 Department of Psychological and Brain Sciences, University of Massachusetts, Amherst, Massachusetts, United States of America; University of California, UNITED STATES

## Abstract

**Background:**

Despite access to free antiretroviral therapy (ART) for all people living with human immunodeficiency virus (HIV), noncompliance to treatment continues to be a significant challenge in Eswatini. Yet studies investigating barriers to ART adherence in Eswatini are scarce. Most notably, there is a lack of research regarding rural women in Eswatini, who are currently the country’s most vulnerable to HIV infection. Therefore, the objective of the study is to investigate individual, household, and community level barriers to ART adherence among rural women living with HIV.

**Methods:**

We conducted a qualitative study to investigate individual, household, and community level barriers to ART adherence. We conducted focus group discussions with HIV-infected women (n = 4) from rural villages in Eswatini, and in-depth interviews with healthcare workers (n = 8) serving the area clinics. Open and axial coding techniques were used for data analysis and interpreted within a social ecological framework.

**Results:**

Our findings revealed several individual level barriers including hunger, side effects of ART, personal stress, lack of disclosure of HIV status, alcohol use, and forgetting to take ART. Lack of food, unemployment and scarcity of financial resources were identified as critical barriers at the household level. Community and institutional barriers encompassed factors related to health delivery such as lack of privacy, travel time, transportation costs, excessive alcohol use by healthcare workers, maltreatment, public and self-stigma, gossip, and long waits at clinics.

**Conclusions:**

Rural women living with HIV face multilevel barriers to ART adherence. Support programs aimed at increasing ART adherence among this vulnerable population need to develop targeted polices to alleviate challenges rural women face, beginning with expanding qualifications for food assistance programs.

## Introduction

More than four decades into the HIV/AIDS crisis, an estimated 37.9 million people around the world are infected with HIV, with sub-Saharan Africa accounting for 68% of the global burden[1]. Current evidence suggests HIV prevalence is highest in Eswatini (formerly known as Swaziland) at 27.4%[2], where incidence is estimated at 1.4%, and is nearly twice as high among women as compared to men[3].

The disproportionate impact of the pandemic on women in Eswatini is attributable to multiple risk factors including biological, social, behavioral, and socioeconomic vulnerabilities[4]. Among other factors, poverty has been identified as both a risk factor for HIV transmission and a significant barrier to ART adherence among women in Eswatini[4–6]. Women living in poverty in rural settings are at a greater risk due to gender norms and traditional roles (such as limited access to education and financial resources) which often forces them to be dependent on men[4–6]. Poor and uneducated women frequently resort to transactional sex to financially support themselves, and many young women have sexual relationships with older men in exchange for money or gifts[4,5,7]. Poverty and gender disparities increase HIV transmission by reducing women's control over safe sex practices, and may negatively impact HIV-infected women’s access to care and treatment[4–6].

Treatments for HIV can improve immune functionality and decrease morbidity and mortality, offering hope that HIV/AIDS can be effectively managed[8,9]. For antiretroviral medications (ART/ARVs) to work effectively and efficiently in preventing the risk of HIV transmission and progression to AIDS, they must be taken consistently as prescribed[8,9]. Patients must be routinely monitored and optimal adherence achieved in order to reach and maintain viral load suppression[8,9]. An optimal level of adherence to ART (taking medication as prescribed with no lapses in treatment) has been shown to be one of the vital components of achieving viral load suppression and avoiding viral rebound among HIV positive individuals[10]. In a quantitative study of ART patients in Uganda, researchers found a 25% increase in odds of viral rebound among individuals who experienced adherence interruptions[11].

In Eswatini, the government provides free antiretrovirals and clinical treatment to all HIV positive individuals, with an estimated 84% of citizens living with HIV receiving medication[12]. Over the past decade, the Ministry of Health, in collaboration with non-governmental organizations (NGOs) and the private sector, has implemented strategies to increase ART coverage and decentralization of HIV care services to regional health facilities, especially in rural areas. Despite this high level of access to treatment, the Ministry of Health in Eswatini identifies nonadherence to HIV treatment as a critical challenge to managing the HIV pandemic[12].

Epidemiological studies have reported high levels of nonadherence to ART in sub-Saharan Africa, with rates ranging from 17.1 to 46% among countries in this region[13–16]. A study conducted with HIV-infected pregnant women in Eswatini reported ART nonadherence to be 50%[17]. Empirical studies across settings in sub-Saharan Africa indicate that ART adherence can be influenced by risk factors operating at multiple levels. At the individual level, ART adherence is associated with age, gender, educational level, forgetfulness, ART duration, alcohol use, stress, lack of money, and lack of disclosure status[17–21]. At the household level, ART adherence is associated with household food insecurity, household socioeconomic status, lack of family support, and use of traditional medicine[18,20,22–24]. At the community/institutional level, ART adherence is associated with area of residence, stigma, lack of privacy and confidentiality, proximity to a health facility, transportation to a health facility, insufficient health care, and maltreatment at health facilities[17,25–27].

From a public health perspective, achieving and maintaining optimal adherence among treated individuals is crucial for the prevention of new infections. Currently, the percentage of patients remaining on treatment after ART initiation tends to decrease over time (i.e. as duration on ART increases, ART retention rates decrease)[28]. Declining retention rates and nonadherence to ART pose significant challenges to HIV treatment and management, particularly for women in their childbearing years since it can negatively impact the prevention of mother-to-child transmission of the disease[29,30].

Seeking strategies to help those individuals that have problems adhering to treatment, the Eswatini government recently implemented community-centered ART service delivery models. Known as Comm ART, this service is aimed at increasing access to ART and improving retention rates for all HIV positive patients, particularly those in remote areas[31]. However, for these programs to be successful in ensuring treatment adherence and patients’ retention to care, it is critical to investigate and identify factors that may facilitate or inhibit patients’ access and utilization of treatment[32]. In an effort to eradicate HIV, Eswatini has adopted the UNAIDS 90/90/90 targets to ensure that 90% of HIV positive individuals know their status, 90% of those eligible receive ART, and 90% of those on treatment achieve viral suppression. These new targets are aimed at helping to reach the government’s goal of an end to new AIDS infections by 2022[28], and to reach this goal, near perfect adherence would be required[33].

Even though ART has been provided for free to all HIV-infected individuals for decades, nonadherence to treatment continues to be a significant challenge for local health facilities, and Eswatini remains the country with the highest HIV prevalence and AIDS mortality rate in the world[2]. Data on ART adherence and its associated barriers in Eswatini is limited, particularly for women living with HIV in rural settings. This study seeks to identify individual, household, and community level barriers to ART adherence among rural women in Eswatini. Findings from this study address barriers to ART adherence in a comprehensive manner, and help us understand the challenges women face regarding access and utilization of ART treatment.

## Materials and methods

### Study population and setting

This study was conducted in Eswatini, a small (17,364 km^2^) country in sub-Saharan Africa. Eswatini has an estimated population of 1.34 million, 63% of which resides in rural settings[34]. The country is divided into four main geographic regions: Hhohho, Manzini, Shiselweni, and Lubombo. The study was conducted in collaboration with four health facilities located in the Lubombo and Shiselweni regions. Two of these facilities are government funded and two are primarily funded by charity organizations. All four facilities collaborate closely with numerous international NGOs. These clinics serve approximately 1500–1700 patients each. Three of the four facilities provide counseling services related to HIV diagnosis, medical care, and ART at no cost to their patients. The remaining clinic provides similar services but charges a small fee (R5.00, approximately $0.40 US) per visit.

#### Treatment and care management

Patients initially diagnosed with HIV are enrolled in lifelong care and evaluated on a regular basis[10]. During the first 6 months after initiating ART, patients are instructed to attend monthly clinic visits to monitor their status and to retrieve medication[10]. Patients are considered stable if they have been on ART and not missed clinic appointments for at least a year, and have undetectable viral loads from their most recent consecutive tests (with the most recent test taken in the past six months) [31]. Stable patients are enrolled into a Comm ART program in which they can receive 3 months’ supply of medication per visit at local clinics or Comm ART centers. Unstable patients are required to continue monthly assessments at the clinic[31]. Adherence, measured through pill count, is assessed every time a patient receives medication either at the clinic or during Comm ART visits. For adherence assessments, patients must bring their remaining medication to each visit for counting by the clinic staff or expert clients. Patients who miss doses frequently (e.g. 2–3 times per week) are provided with counseling sessions intended to motivate consistency in medication adherence[10].

### Study design

**Fig 1:**
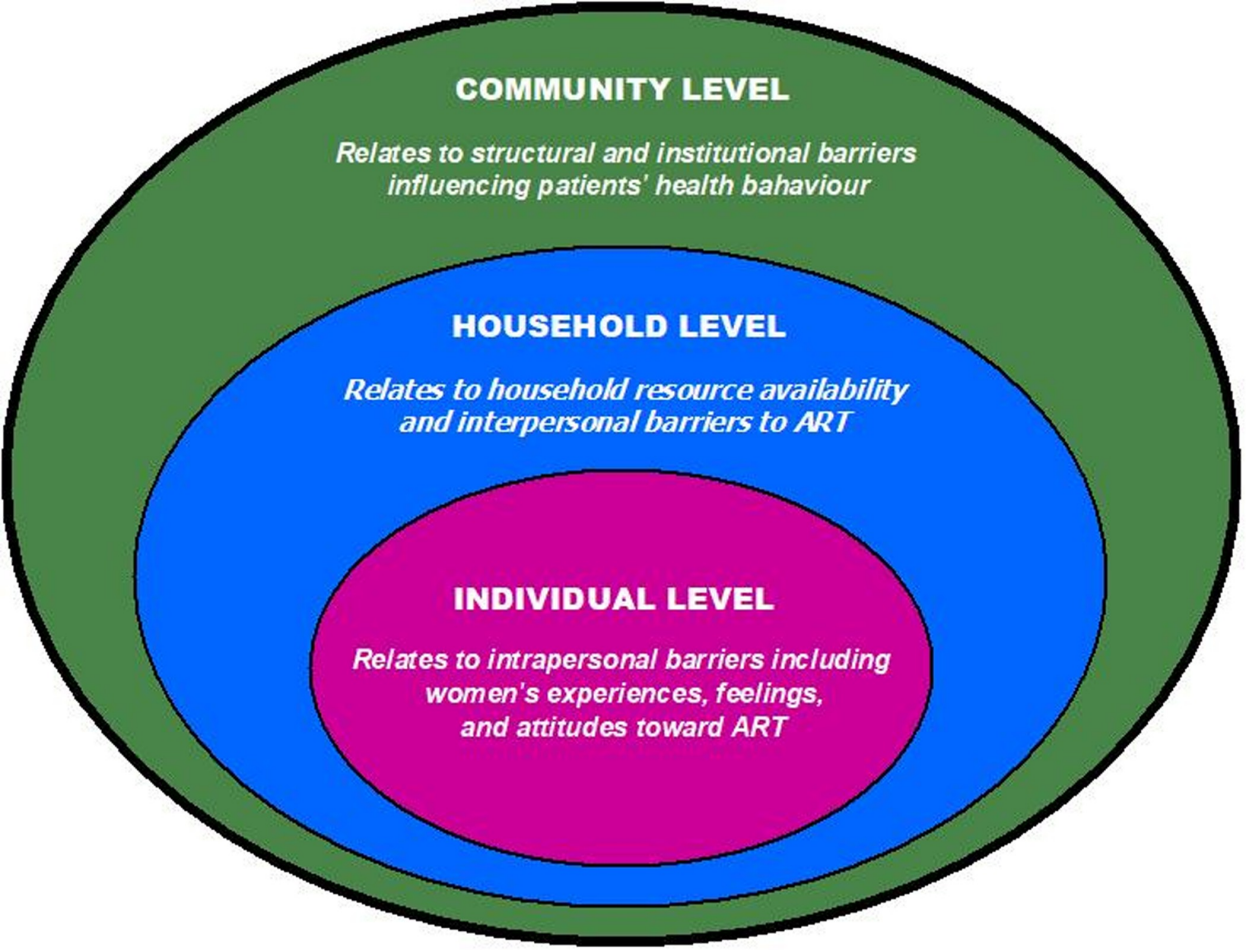
The social ecological model[39].

We conducted a qualitative study to investigate barriers to ART adherence among women living with HIV in rural Eswatini. This study is part of a larger study conducted between May and November 2017. Here we report on the qualitative part of the study. Health behaviors are better understood with the use of a theoretical framework that takes into consideration complex ecological dynamics and their interaction with individual factors[35,36]. This study uses a social ecological model (SEM) to explore how factors across multiple levels interact and influence ART adherence (Fig 1). The SEM is used in public health to understand the manner in which factors within multiple environmental domains exert an influence on individual health behaviors and outcomes[13,37,38].

#### Eligibility criteria

The study population consists of rural Swazi women receiving HIV antiretroviral therapy. Women were eligible to participate in the study if they had been on ART for at least a month (at the time of the focus group discussions), were between ages 20–49 years, and were willing to provide written informed consent.

#### Sampling and participants recruitment

We employed a convenience purposeful sampling technique to recruit participants into the study[40]. Participants were recruited in collaboration with local health facilities and community health workers or expert clients. The research team, which consisted of the Principal Investigator (PI) and two research assistants (RAs), visited the health facilities and worked with community health workers to identify target communities. Once target communities were identified, the research team attended community outreach activities and support group meetings in those communities where they introduced the study to potential participants. Women who were interested were invited to participate in focus group discussions (FGDs). In addition, the research team employed a snowball sampling technique and worked with local community health workers (expert clients) to recruit other qualifying individuals to participate in the surveys. During recruitment, it was emphasized that participation in the study was entirely voluntary, that all interviews were confidential, and patients’ willingness to participate would have no effect on their treatment at the clinic. Participants were compensated with a R100 for traveling expenses and for time taken to participate in the study. Healthcare workers (including nurses and expert clients) were recruited as key informants, using purposeful sampling [41].

### Data collection measures

We conducted four FGDs (8–12 women per group) and in-depth interviews of key informants (8 healthcare workers) in June 2017. Key informant interviews were conducted at the clinic while FGDs were conducted in a central location within respective rural communities (such as local community buildings, churches, or neighborhood care points). Key informant interviews were used to gain insight concerning what front-line healthcare workers observed as barriers to ART adherence. Key informants were asked to reflect on their observations and perspectives regarding barriers to ART adherence in their respective communities. Written consent (in the local language of siSwati) was obtained from participants at the time of the interviews or FGDs. The research team read and explained the consent form passed out to study participants. During FGDs, participants were provided time to ask questions or clarify any content of the consent form, and if they agreed to participate in the study, were asked to provide signed consent. The FGDs participants were asked to provide demographic data including: age, area of residence, marital status, and educational background. Many of the women were illiterate and unable to write down answers, so the field team helped them enter demographic information on the forms whenever necessary.

The purpose of the focus group was to collect in-depth data on women’s lived experiences, focusing on barriers that impacted their ART adherence. While using FGDs to document personal lived experiences is uncommon in qualitative research, due to the sensitivity of the subject matter and the stigma associated with HIV in these communities, we were advised by local health workers that one-on-one interviews would likely produce guarded answers and that FGDs would allow the women to speak more freely. Focus groups were conducted in siSwati by the PI (Becker) using a moderator’s guide consisting of semi-structured questions to facilitate group discussions, while encouraging discussion of emergent relevant and related topics. Specifically, participants were asked to reflect on the following: 1) individual, interpersonal, social, and structural barriers to ART adherence, 2) feelings about and experiences with ART, 3) challenges in ART service provision, 4) social support received from friends and family, and 5) personal beliefs and societal norms as they relate to ART. Observations concerning impressions or insights that involved the research setting, group dynamics, or non-verbal communications witnessed by the research team in these discussions were noted. The FGDs were digitally audio-recorded. The recordings were transcribed verbatim in siSwati and then translated to English. After transcription, data was encrypted and stored on a password protected computer. Measurement instruments (interview and FGDs moderator’s guides) were pilot tested prior to conducting this study.

### Ethical clearance

Study approval was obtained from the University of Massachusetts Amherst Institutional Review Board and the Eswatini National Health Research Review Board. Permission to conduct the study was also obtained from the Eswatini Ministry of Health and participating health facilities.

### Data analysis

Data was analyzed using the constant comparative method[42–44]. Phase one of data analysis consisted of each member of the field team (PI and two RAs) independently coding all data sources (key informant interview transcripts, transcripts of FGDs, and observational notes) using Nvivo version 15[42,43]. Coding consisted of reviewing of transcripts line-by-line to isolate key words and phrases identifying barriers to ART adherence then using an open coding approach to generate emerging themes and nodes[42,43]. As identifiers became apparent, codes linked to associated nodes were assigned to each incident of the word or phrase in the source documents.

In the second phase of the analysis, the PI and three RAs reviewed divergent and convergent emerging themes, reaching consensus on themes. An initial codebook was created, containing clear definitions for each code with examples of their application within the transcripts. Next, the PI and a fourth RA independently applied the codebook to a smaller set of transcripts and reviewed for consistency. This process was carried out twice again, first with a previously unused set of transcripts and then with the full set of transcripts. Coding inconsistencies were resolved through research team discussions and the codebook was adjusted accordingly.

Finally, data was triangulated (adding observational notes) to ensure consistency and ascertain validity of the findings. Employing the process of inter-coder agreement, the research team did a side-by-side comparison of all coded material, again to identify convergent and divergent findings[43]. Using the codebook, we thoroughly reviewed all coded text passages to determine whether codes assigned to text passages were similar or different. Coding inconsistences were resolved through team discussions and adjusted accordingly. Coding comparison queries were used to develop percentages of codes that were either similar or different, and reliability statistics (kappas) were computed for systematic data comparisons[43].

After this step of analysis, the coding team applied a deductive approach to the data whereby codes were grouped into themes and related to the social ecological model. This was done through axial coding, where the main themes that emerged from open coding of the data were interconnected with each other and interpreted using the social ecological model[42].

## Results

Data for this study was collected from FGDs (n = 4) of rural women receiving ART and in-depth interviews with healthcare workers (n = 8). Coding comparison queries were used to determine regularities, with a majority of the identified themes (96.09–100%) found to be consistent across coders. Interrater reliability statistics (kappas) were computed for systematic data comparisons, and obtained acceptable reliability coefficients ranging from 0.72–0.99 across all identified themes[42,45]. The mean age of the focus group participants was 36 years and almost half (n = 19) were married (Table 1). While a majority of the women (n = 32) had some primary or high school education, 7 had no formal education. None of the women had attended college. Most of the women (n = 36) had occasionally obtained food and/or financial support from neighbors, friends, or family members. All the women interviewed reside in impoverished, drought-stricken communities.

**Table 1:** Respondents demographic information.

Characteristics	*N*
Mean Age	36
**Education**	
Did not attend	7
Primary	16
Secondary	16
**Marital Status**	
Married	19
Not married	13
Living with partner	2
Divorced/separated	2
Widowed	5
**Support**	
Family	32
Friends/neighbors	4
No one	5

**Fig 2:**
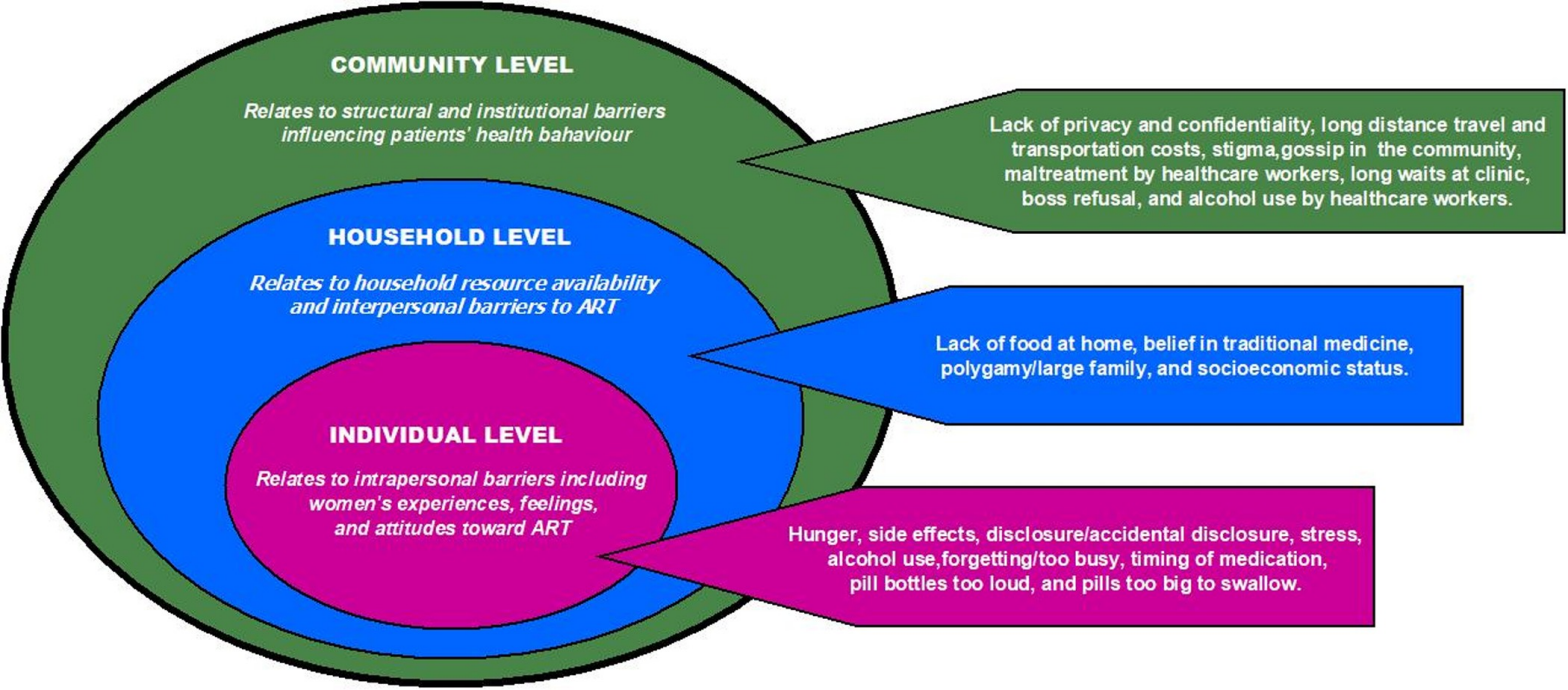
Summary of themes identified based on the social ecological model.

The study findings of emergent themes are presented using a social ecological model to explore and understand the multiple level factors that interact and influence ART adherence (Fig 2). In this section, we report barriers influencing ART adherence at the individual, household, and community levels, respectively. The individual level identifies intrapersonal influences of ART adherence, including women’s experiences, feelings, and attitudes towards ART. The household level examines the household environment and resource availability, social, and interpersonal factors (including relationships with various persons or groups such as family, friends, spouses, and sexual partners) that act as barriers or facilitators of ART adherence. The community level examines structural and institutional barriers that influence women’s health behavior including access and utilization of ART.

### Individual level factors

Primary factors identified as barriers to ART adherence at the individual level included: hunger and hunger-related medication side effects, stress, lack of disclosure or accidental disclosure, being unsure of time to take medication or forgetting to take pills, loud rattling of pills in their bottles, and pill size too big to swallow. Excess alcohol use was identified by healthcare workers as a negative influence on ART adherence (Table 2).

**Table 2:** Individual level barriers to ART adherence.

Identified Barriers	Individual level barriers as reported by study participants	
	Patients	Healthcare workers
Hunger and hunger-related medication side effects	*“You need to eat first*. *I once fainted when I took the pills without eating*. *I take it twice a day*, *morning and evening*. *Sometimes I skip taking it if I don’t have anything to eat*.*” (Patient S)*	*“Most people say they stopped taking ARVs because they didn’t have money to buy food*. *They complain that the medication make them sick if they take it hungry*.*” (Healthcare worker C)*
Lack of Disclosure/Accidental Disclosure	*“Even though refills are done at …*, *I like coming here to …. because nobody can see me*. *Even though I accepted my situation*, *I don’t want other people to know because people talk*.*” (Patient S)*	*“And you find that when we call the number they gave us*, *the person on the phone either doesn’t know the client or they are not aware that they’ve been registered as the patient’s treatment supporter*. *Sometimes it’s very difficult to reach them*, *especially those who haven’t disclosed…they run away and hide from us… they don’t like home visits*.*” (Healthcare worker S)*
Timing of medication/ Forgetfulness	*“I don’t work either but I run a vegetable stand*. *Sometimes I walk around looking for avocados like now its avocado season*. *I may get busy*, *come home late*, *and forget to take my pills on time*.*” (Patient S)*	
Alcohol use	*Oh my child*, *people drink in this community*. *They drink a lot*. *They even take the ARVs with alcohol*. *And sometimes they get so drunk and don’t take their pills*. *(Patient G)*	*“…alcohol use plays a role*. *Alcohol makes them forget the dates for clinic appointments*. *I also think that it makes them forget taking their medication on time at home*.*” (Healthcare worker S)*
Pill size too big to swallow and make noise	“…*also that the containers make a lot of noise*. *Sometimes I can’t go to the clinic*, *so I ask my sister to pick up the pills for me*. *She says*, *“hey you know those pills are too loud*, *when I get off the bus I need to carry my bag with care because they make a lot of noise*.*” (Patient C)*	*“…some people still have stigma of being found out that they’re taking ARVs*. *They don’t want to be seen carrying the pills*, *they say people can hear them because the pills make noise*. *They often take them out of the container and put them in the regular plastic packets*.*” (Healthcare worker M)*
Stress	*“Problem I encounter is that I am unemployed so sometimes something happens*, *I have too much stress and forget taking my pills*.*” (Patient C)*	

#### Hunger and hunger-related medication side effects

Hunger and hunger-related medication side effects were often reported by both healthcare workers and women as a significant barrier to ART adherence. A majority of the respondents reported the main reason they missed doses was lack of food, and that they were afraid of getting sick if they took the medication on an empty stomach. This was particularly challenging for women living in poverty who reported occasionally going several days with insufficient quantities of food to feel safe taking the medication. Some of the women reported that medication side effects were so severe that they had difficulty sleeping at night and often struggled to function normally during the day. Healthcare workers also reported lack of food as a significant barrier to ART adherence. They pointed out that due to high unemployment rates and five consecutive years of local drought, many of their patients were struggling to make ends meet, with lack of food being a primary concern.

#### Lack of disclosure/accidental disclosure

Women regularly conveyed fear of having their HIV status revealed to family or community members. Though nearly all study women had disclosed their HIV status to at least one person (typically a close friend or family member), the women were concerned about accidental disclosure of their status to members of their social group and/or communities. Some women reported experiencing conflict with spouses or partners after disclosing their positive HIV diagnosis. Furthermore, women reported fear of being seen near the mobile clinic during Comm ART visits, and worried about being recognized at the clinic’s ART unit when picking up meds or attending appointments. Some even opted to travel to more distant clinics to avoid being seen by people they know at the facility. Healthcare workers corroborated the above comments, adding that they suspect the reported level of HIV status disclosure may be overestimated. They reported finding that often patients would provide false contact details regarding their treatment supporters. It was suspected that many patients likely had not disclosed their HIV status to anyone.

#### Timing of medication/forgetfulness

Uncertainty regarding time of day was another reason cited for missing medication. Some women reported taking pills late because they did not have a clock, radio, or cellphone to help them keep track of time. They stated that frequently asking “What time is it?” to family or community members put stress on those relationships, expressing concerns that they were also increasing the risk of revealing their positive status. Some of the women reported that they sometimes forgot to take their medication due to being too busy, being away from home, stress, alcohol use, or having to attend family or community events (e.g. all-night vigils for local funerals, a traditional mourning practice).

#### Alcohol use

Excessive alcohol use was identified by participants as a major barrier to ART adherence. During the discussions, we found that most women were apprehensive about discussing their own alcohol use. They spoke about alcohol use in very general terms, identifying the behavior as a barrier to adherence but were unwilling to disclose specifics regarding their own alcohol consumption practices within the focus group setting. However, during the interviews with healthcare workers, excessive alcohol use emerged as the most frequently cited reason for HIV-infected women missing their ARV doses. Additionally, healthcare workers reported that patients’ alcohol use often led to missed clinic appointments.

#### Pill size too big to swallow and make noise

Some women reported having difficulty taking their medication due to the large pill size. This was particularly challenging for those who took morning and evening pills. They reported that even if they took them with plenty of water as per other participants’ suggestion, the pills got stuck in their throats causing gagging and sometimes vomiting. A majority of the women complained about the loud rattling of the pills in the containers. They reported that the pill containers are very noisy and make a discernable sound that would be recognized as ARVs. They felt embarrassed by the sound and it made them not want to bring pills along while traveling on crowded buses (the main form of transport). Healthcare workers reported patients regularly complaining about the sound the pill bottles make.

#### Stress

Feelings of stress were described by some of the women as a significant barrier to ART adherence. Primary sources of stress involved anxiety over food supply and lack of financial resources to provide for their children. They reported sometimes forgetting to take pills due to emotional or stressful life events. Some women reported drinking alcohol as a way to cope with stress and hunger.

### Household level factors

Lack of food at home and limited financial resources were identified by women as primary barriers to ART adherence at the household level. Health care workers mirrored those concerns, reporting that tolerance of polygamy within households was also having a negative effect on ART adherence (Table 3).

**Table 3:** Household level barriers to ART adherence.

Identified Barriers	Household level barriers as reported by study participants	
	Patients	Healthcare workers
Lack of food at home	*“The painful thing is that we don’t have any food to eat at home*. *When I take my pills hungry*, *I get severe headaches and burns in my stomach*.” *(Patient S)*	*“…some report lack of food*. *They say they can’t take the pills if they haven’t eaten*.*” (Healthcare worker M)*
Socio-economic status	*“I don’t have the money for bus fare to pick them up at the clinic* … *You find that the date to go to the clinic arrives and you don’t have money*.*” (Patient C)*	*“Some have reported lack of money for transport yet they don’t want to use our community ART program* …*” (Healthcare worker G)*
Polygamy and large families	*“Another thing is the issue with the husbands*. *You find that you go get tested and find that you are positive and your husband is not*. *Once he finds out he’s negative*, *he comes back and accuses you of sleeping around*. *They ask too many questions and accuse you of cheating*. *This is giving us problems because you find that there’s no support and sometimes he tells you that he doesn’t trust you and will never trust you because he doesn’t understand where you got the HIV from because he doesn’t have it*.*” (Patient G)*	*“I think living in big families does play a role*. *Sometimes wives are afraid to tell their husbands because they worry that if they tell them the husband will tell the other wives and everybody will blame her for bringing HIV to the family*.*” (Healthcare worker C)*

#### Lack of food at home

Study participants reported shortage of food within the household as the primary reason individuals default on treatment. In an effort to prolong household food supply for the whole family, some women reported limiting personal meal frequency and reducing portion sizes, sometimes skipping meals entirely for one or more days to ensure that their children had something to eat. Food insufficiency in the household made it difficult for the women to prioritize treatment adherence on a daily basis, particularly those experiencing adverse hunger-related medication side effects.

#### Socio-economic status

Poverty and unemployment are factors that were commonly reported by the women and healthcare workers as having a critical negative influence on adherence. The women in our study stated that because they were unemployed, they struggled to provide basic needs for their children (food, clothing, school fees, etc), often did not have bus fare to attend clinic visits, and found it difficult to eat enough before taking medication. Women with insufficient financial resources reported being faced with difficult choices such as whether to spend what little money they had on food for their children, or to travel to an appointment at the clinic for HIV treatment and care.

#### Polygamy and large families

Polygamy was believed by healthcare workers to negatively affect adherence. Healthcare workers reported that many patients in polygamous or large families chose to hide their HIV status due to fear of being rejected and ostracized by family members. This created problems with taking their medications consistently. Women in polygamous households who had disclosed their HIV status to their husbands reported fearing that co-spouses who would then judge and blame them for bringing disease into their families.

### Community level factors

Community level barriers to ART adherence commonly reported by participants included lack of privacy at the clinic, travel time, transportation costs, public and self-stigma, gossip in the community, maltreatment by health workers, and excessively long waits at clinics. An unexpected but commonly reported barrier regarded local employers not allowing workers to take time off for clinic visits. Health workers reported that excessive alcohol use among co-workers while on duty was having a negative influence on patient’s ART adherence (Table 4).

**Table 4:** Community level barriers to ART adherence.

Identified Barriers	Community level barriers as reported by study participants	
	Patients	Healthcare workers
Stigma and gossip	*“You also find that you’re worried that if you tell people you live with about your situation*, *they will go around telling everybody in the community…*.*They gossip about people in the community*.*” (Patient S)*	*“…people gossip about them*, *they say all kinds of negative things about people taking ARVS*. *They even use derogatory terms like “phinduvuke (rose from the dead)*, *hohlohohlo (rattle sound made by ARVs pill bottles)*.*” (Healthcare worker G)*
Employer refusal	*“Like they’ve said it’s a problem at work*. *When I asked to come to the clinic from my boss*, *he told me to leave and never come back…*. *He refused and said if I leave I leave for good*. *I stayed…*.*” (Patient S)*	*“But another big problem here at* ….*are the firms*, *their bosses don’t want them to take a day off to come pick-up their meds every month*, *even if we give them a sick note*.*” (Healthcare worker M)*
Long distance travel and transportation costs	*“We miss the date for clinic visits if there’s no money for bus fare*. *It passes and they yell at me*.*” (Patient C)*	*“…traveling distance and lack of bus fare to the clinic makes it difficult for people to get their pills*.*” (Healthcare worker C)*
Maltreatment by healthcare workers	*“They don’t understand*, *they yell*. *One time I missed the date for clinic visit and they made me sit outside for hours…*. *I thought of leaving but I couldn’t because I knew I was going to go through the same thing if I left*.*” (Patient C)*	*“…poor service at the clinic might be driving some people away*. *When they come here looking for help and they’re not treated well by the staff*, *they go away and don’t come back*, *sometimes for months on end*.*” (Healthcare worker S)*
Lack of privacy and confidentiality	*“Problems we encounter are problems at the hospital*. *Because sometimes you get scared to go pick the medication*, *because when you get there sometimes*, *they give you the green files to hold while waiting and everybody can see*.*” (Patient M)*	*” Some stop coming to the clinic altogether because they don’t want to be seen*.*” (Healthcare worker G)*.
Long waiting time	*“Even at 4 when they close*. *They leave you like that*. *Even if you can wake up early* … *like at work you ask permission from the boss thinking that you’ll get helped early but no*. *Then you miss work*, *and they don’t give you a sick note…then it’s a problem now*.*” (Patient M)*	*“Some people complain about the waiting at the clinic*. *We are understaffed here so sometimes it takes a long time to help everybody*.*” (Healthcare worker S)*
Alcohol use by healthcare workers		*“… health workers themselves*, *the people you expect to counsel them against drinking and to lead by example*, *are drunkards themselves*. *…we tell them to not drink alcohol because it makes the medication not work properly*, *but how can you expect them to take us seriously when some health workers are doing the same*.*” (Healthcare worker G)*

#### Stigma and gossip

Public stigma (real or perceived) and self-stigma were reported as some of the reasons women missed treatment dosage, missed clinic visits, or discontinued ART treatment. Most women had a treatment supporter (presumably an individual who was aware of their HIV status), but many had concerns about disclosing their HIV status to families, communities, or social networks for fear of being scorned or rejected. Some reported having heard negative gossip about individuals receiving ART, and worried that individuals in their families, communities, and social networks would think less of them if they knew they were taking “bophinduvuke/ bohohlohohlo” (derogatory terms for ARVs), and they would also become the source of gossip. Healthcare workers echoed patient’s concerns and acknowledged gossip was still widespread in the communities that the clinics served. They reported that due to fear of being the subject of gossip, patients would often avoid Comm ART services, fail to attend clinic appointments, skip doses, and neglect refills.

#### Employer refusal

Most of the employed women reported that they did not disclose their HIV status to their employers and co-workers. Several women talked about occasionally missing clinic appointments or failing to refill medication due to a boss or supervisor at work refusing to give them time off. When they ask to attend a clinic appointment, supervisors demand a reason for going, which resulted in fear of disclosing status and being overheard requesting to pick up HIV medication. Some of the women also experienced threats of job termination by their boss/supervisor when they requested time off to attend a clinic appointment. Healthcare workers reported similar concerns.

#### Long distance travel and transportation costs

Transportation costs and travel distance were reported by the women and healthcare workers as contributing factors to nonadherence. For women living in remote rural villages, just getting to the clinic to pick up monthly prescriptions poses a significant challenge, often taking the entire day. Women with limited financial resources frequently reported being unable to afford the bus fare to the clinic. Many were forced to walk long distances to and from the clinic (10 miles or more). Several women reported that if they did not show up early enough to the clinic, they risked not being seen that day and would have to repeat the travel process for a rescheduled appointment. Some described having to wake before dawn and walk several hours in order to arrive at the clinic by 8am. These financial and geographic challenges led to missed clinic appointments and poor adherence to ART.

#### Maltreatment by healthcare workers

The women reported being treated poorly by clinic staff, describing that they would get yelled at or punished if they arrived at the clinic late (after 11am) or missed an appointment. Furthermore, the women stated legitimate excuses for missed appointments were never accepted, and when they did attend the clinic at a later time, healthcare workers would humiliate them and make them wait outside until everyone else had been helped. Women also described being reprimanded at pill counts if they had the incorrect number of pills left. As a consequence, they reported throwing away excess pills prior to counts, to ensure that they would not be treated poorly. Healthcare workers acknowledged that there were problems with the way patients were treated by clinic staff. Those interviewed stated that healthcare workers often yelled at patients, using derogatory terms such as “laba babo ART” (those for ART) when referring to patients receiving treatment.

#### Lack of privacy and confidentiality

Lack of privacy and confidentiality at the hospital was reported as a major barrier to adherence by both the women and healthcare workers. Some patients complained that local healthcare workers lacked confidentiality as they would sometimes gossip or discuss patients’ HIV status with other community members. The women also expressed anxiety regarding the separate and distinct location of ART Units within the clinics. They spoke of feeling uncomfortable being seen while picking up easily identified ART pill bottles from the pharmacy window. They also complained about having to hold their distinctly colored HIV Care files while queuing (usually in open spaces) for appointments or moving around the clinic, which compounded concern of being identified by family and/or members of their community. Healthcare workers corroborated the women’s concerns and elaborated that for some patients the lack of privacy and risk of exposure was so unbearable that they stopped going to the clinic entirely.

#### Long waiting time

Long wait times at clinics were identified by both healthcare workers and the women as a major problem, particularly for people rushing to go back to work and those living in remote areas. The women reported having to wait long hours for service at the clinics. Some reported waiting up to six hours before receiving care. At times, the wait was so long that the clinic closed (at 4pm), and they were forced to leave without medication refills. The wait was particularly difficult for employed individuals, risking losing wages and at times even their jobs.

#### Alcohol use by healthcare workers

During interviews, healthcare workers identified excessive alcohol consumption by co-workers while on duty as a major barrier to ART adherence. They described some co-workers arriving at work at 8am already intoxicated, while others would consume alcohol during lunch breaks. They believed that co-workers’ alcohol consumption negatively impacted service delivery and was affecting ART adherence.

## Discussion

Our findings reveal that nonadherence to ART is a complex behavior that is heavily influenced by barriers operating at individual, household, and community levels. We found that hunger resulting from household food insecurity and low socio-economic status had a negative impact on ART adherence due to adverse side effects from medication taken without food. In our study, reports of portion size reductions and decreases in meal frequency were common when household food quantities were limited. This often led to lapses in ART adherence. In recent qualitative and quantitative studies conducted among countries in sub-Saharan Africa, hunger and household food insecurity were identified as major barriers to ART adherence [20,21,23,46–48]. Several studies in the region have reported similar findings, and in some cases, individuals stopping treatment completely due to anxiety over side effects from ART medication when access to food was compromised and inadequate [18,19,27].

The communities covered in our study suffered from widespread poverty exacerbated by drought, and during the timeframe of our study, they received no nutritional support from government or NGO sponsored programs[49]. Intervention studies providing food supplements and nutritional support have been shown to improve ART adherence[50,51]. In a recent World Food Program report of the Food by Prescription program in Eswatini, food supplementation was shown to significantly improve ART adherence[52]. Findings from this and other studies strongly suggest that the implementation of sustainable nutritional and food security interventions integrated with HIV/AIDS programming would substantially improve ART adherence[50,51]. An expansion of food and nutrition programs for all HIV-infected individuals would be beneficial for ART adherence, and would likely improve general health outcomes, reducing overall healthcare costs[30].

In our study, stress due to lack of money or food was identified as a barrier to ART adherence, and was reported as the main reason for alcohol use in these communities. In addition, alcohol use was frequently cited as a typical reason patients forgot to take medication, and why they missed clinic appointments. Alcohol use has been identified as a contributing factor to ART nonadherence among countries in southern Africa[19,20]. In a qualitative study of HIV positive adults in South Africa, some patients confessed to discontinuing treatment due to regular alcohol consumption[20]. Parallel findings were reported in a study investigating barriers and facilitators of ART adherence among HIV positive patients in Lesotho[21].

Our study indicates a need to intensify strategies aimed at identifying and addressing the root causes of stress for people living with HIV. The communities covered in our study would benefit from programs designed to reduce poverty and unemployment (such as improved access to education and economic opportunities for women), and to provide access to counseling and alcohol rehabilitation therapy. Implementing community outreach programs aimed at educating patients regarding the effects of alcohol on ART and general health, and providing alcohol rehabilitation centers in rural communities may help increase awareness and reduce alcohol consumption among people living with HIV.

Patients reported that confidentiality issues and a general lack of privacy had a significant influence on their ART adherence. Other studies have reported lack of confidentiality as a major reason patients missed clinic appointments and discontinued accessing ART from local clinics[25,37,53]. In our study, women expressed concerns about the location of the ART units and/or pill counting stations at the clinics, and feared that they would be seen by people they know while queuing for service during clinic visits. In a recent study conducted among caregivers of HIV positive children in Eswatini, researchers reported that in an effort to gain privacy, patients routinely switched clinics, which led to treatment interruptions and missed clinic appointments[37]. Perhaps changes should be made to the visibility of ART units with an emphasis placed on privacy and confidentiality. Patients should not have to carry color coded medical record folders which reveal HIV status. For example, in order to improve privacy and confidentiality, patients’ medical records folders should be discreetly transported to consultation rooms by clinic staff. Another common privacy concern involved the rattling noise made by ARV pill bottles. To address this issue, containers could be altered to reduce noise, or an alternate disbursement method could be employed. For instance, ARVs could be packaged in plastic pill packets (currently a standard medication disbursement method at clinics) which could help prevent accidental disclosure of HIV status.

Women in our study were concerned about a lack of professionalism among clinic staff. Poor treatment by healthcare workers (yelling at patients, punitively punishing patients, unnecessarily long wait times, revealing HIV status, gossiping about patients) was reported by many as a contributing factor to nonadherence. Maltreatment by healthcare workers may discourage patients from returning to the clinic for their refills and may lead to patients being lost to follow-up and defaulting treatment[37,53]. In a study investigating patients’ attitudes toward decentralization of HIV care in South Africa, fear of maltreatment by staff was reported as a major concern among patients accessing ART from local clinics[25]. Initiatives are needed to retrain healthcare workers regarding professional conduct, sensitivity towards patients’ needs, and confidentiality of patients’ medical records. Studies have shown that a good relationship between healthcare workers and patients facilitates adherence[54,55]. Prioritizing a healthy patient-staff relationship could help improve service delivery and retention of patients in care.

Gossip (at the individual, household, and community levels) is rampant among rural communities of Eswatini, and being the subject of gossip is regarded as an egregious insult to one’s character[56]. In our study, patients worried that if people became aware of their HIV status, they would become the subject of gossip among family and community members. This behavior can make it harder for people living with HIV to feel accepted. Stigma (both self-stigma and public stigma) at the individual, household, and community levels were often cited as contributors to nonadherence, as patients reported fear of being judged and shunned by people in their families, communities, and social networks if they were known to be using ARVs. Various studies have identified fear of accidental disclosure and stigma as significant factors affecting ART adherence[17,20,25]. In a recent study of HIV positive pregnant women in Eswatini, Gary et al. (2015) examined and described factors influencing ART adherence and found that self-perceived stigma and lack of disclosure were often reported as contributors to poor ART adherence. In order to encourage the utilization of local ART services and reduce treatment default, it is essential to devise strategies at both national and local (chiefdom) levels to eradicate all forms of HIV stigma among the rural population.

Despite the availability of Comm ART services in most communities, transportation cost and travel distance emerged as significant barriers to ART adherence. In resource limited settings, long travel distance to health facilities and transportation costs were commonly cited as barriers to ART adherence[19,20,37]. While the Comm ART service delivery models have the potential to improve access to ART for those in remote areas, only patients who have proven to regularly take their medication are eligible for this program[32]. Patients defaulting treatment would be deemed ineligible for Comm ART services. Perhaps Comm ART should be expanded to additional communities and made accessible to patients of all levels of adherence. This could be of substantial benefit for patients defaulting treatment due to financial constraints, particularly those living in remote areas[57,58]. Transportation costs need to be addressed. No one should be denied access to medication or have to miss appointments simply because they cannot afford bus fare to the nearest clinic. Initiatives aimed at reducing patients financial burden regarding HIV treatment (such as implementing transportation services to shuttle patients to clinics or providing cash vouchers to cover transportation costs) would be most beneficial, particularly to those of low socio-economic status [59].

### Strengths

To our knowledge this is the first qualitative study to investigate barriers to ART adherence among rural women in Eswatini (one of the most vulnerable populations for HIV). As far as we know, our study is the only study to reveal additional barriers such as gossip and alcohol use by clinic staff that has not been reported in previous studies. By describing multilevel factors that exert an influence on patients’ ability to access and utilize ART, we are able to comprehend and contextualize the difficulties that women face regarding treatment. This study utilized a data triangulation approach (concurrently gathering data from patients and healthcare workers) which helped strengthen the authenticity of the reported themes and provided comprehensive understanding of the factors influencing ART adherence in a rural setting. This information may help inform policies and potentially assist in designing and implementing support programs aimed at increasing ART adherence in Eswatini.

### Limitations

The purposeful sampling method used to recruit participants into the study may be limited by a level of selection bias, given that focus groups were comprised of women living with HIV who chose to participate, not all infected women approached participated in the study. It was especially difficult to reach women completely defaulting on treatment, as those women were actively avoiding contact with health workers. Inability to recruit these women could have biased the study sample as people who did not participate may have had additional barriers that were not reported by those that participated. Our study may be limited due to the minimal number of interviews conducted with healthcare workers, however we believe that the information gathered was sufficient to answer our research questions since there were no new emergent themes identified beyond the sixth interview. As with all qualitative research, the role of the moderator or interviewer could potentially be a source of bias. In order to mitigate potential moderator or interviewer bias, all interviews and FGDs were moderated by the PI (trained in qualitative research methods and conducting interviews). While focus groups are useful for understanding experiences and attitudes of a group or personal health behaviors, an inevitable amount of bias could occur through group dynamics. Participants may be reluctant to voice opposing views, or the more assertive participants may disproportionately influence reported themes. However, we believe peer influence was minimal as all participants were encouraged to share their honest opinions and were given an opportunity to discuss their experiences or feelings toward ART freely. Additionally, the data triangulation approach and the process inter-coder agreement employed during data analysis helped strengthen the credibility and dependability of the reported barriers. Despite the limitations, this study provides a better understanding of the individual, household, and community level barriers influencing ART adherence among women in rural Eswatini.

## Conclusions and recommendations

The poorest and most vulnerable HIV patients in Eswatini frequently face challenges that significantly impact ART adherence. We conclude that more emphasis should be placed on interventions to improve food and nutrition security for people living with HIV in order to mitigate the effects of hunger on ART, alleviate medication side effects, and subsequently improve ART adherence. We recommend that the Comm ART program should be opened up to patients of all levels of adherence in order to ensure timely and easy access to medication for women in rural communities, particularly those with limited financial resources. In addition, Eswatini should prioritize implementation of policies to address issues of gossip and stigma. Increasing interventions aimed at growing awareness and acceptance of those living with HIV may reduce patient anxiety toward accessing local ART services in rural communities, and would create a supportive environment whereby adherence is viable for women in Eswatini and other high prevalence settings.

## Supporting information

S1 Data Key informant interview transcription. (PDF)
