# Supplementary material for: Individual, household, and community level barriers to ART adherence among women in rural Eswatini
Source: PLoS One. 2020 Apr 28;15(4):e0231952. doi: 10.1371/journal.pone.0231952 (PMC7188206; doi:10.1371/journal.pone.0231952)
Supplement: S1 Data — (PDF) [file pone.0231952.s001.pdf]

KEY INFORMANT INTERVIEW TRANSCRIPTION

Q 1. Tell me about the women in your community? (Probe for: demographics, atmosphere, what you feel proud about, what are some challenges?)

C 1

Most of the people here are illiterate. When I give them the medication I explain the importance of adherence. I tell them that the pills will help them if they take them truthfully.

Q 2. What is the nature of your usual interaction with the women you serve?

C 1

I often see them after they get seen by the nurse or doctor, then they come to me to get their medication and sometimes they get vitamin supplements (for pregnant women).

Q 3. What are some of the issues you see among women on HAART?

C 2

Yes, there are those who are nonadherent. They report lack of food, and even when you look at them you can see that they're really struggling. When I see someone like that, I tell them to report this to the nurse so that they can get some food rations (corn meal and bean) that may last them for a couple of days. Some people report lack of bus fare as well but not that many, it's mainly hunger.

Q 4. What is your sense about the women's attitude/feelings towards HAART? How about their spouses/friends/family members?

C 1

People around here are very accepting of their HIV status. Sometimes I get amazed how is easy it easy for some people to accept it. Some people are still struggling with telling their family members about it. There's this young lady who keeps asking me to transfer her meds to the plastic packets because she doesn't want people to find out that she's taking ARVs. I do that for them if they ask.

Q 5. What kind of comments or stories do you hear from the women about HAART in general?

C 1

Yes some of them complain about the side effects. I heard another lady complaining that her husband is worried because the pills are making her lose weight. Some people make them fat in weird places. I often counsel them and tell them that it's better to be alive and deformed than dead.

Q 6. What are some of the reasons women give for not taking their medication consistently?

C 2

Some people complain about the lack of money. But you find that sometimes patients would show a 100% adherence but when you look at them, they look so sick. Then you wonder what they do with pills, sometimes I think they throw them away.

Follow up question

Shoktsi le disclosure

Response

Its difficult, especially for the younger ones.

Q 7. Do you think women skip taking their ARVs if they do not have enough food to eat? Do they mention lack of food as one of the reasons for not taking their ARVs?

C 1

(Yes, see Q3)

Q 8. Do you think travelling long distances to the clinic and lack of bus fare make it difficult for women to adhere to medication? Please elaborate.

C 1

Yes, traveling distance and lack of bus fare to the clinic makes it difficult for people to get their pills. You find that people walk long distances to come to this clinic because they like the staff and when we suggest transferring them to closer clinics, they refuse. They say they like it here.

Q 9. Do you think cultural traditions/societal norms (e.g. polygamy) play a role in whether or not women take HAART? If so, how?

C 1

Yes a lot. Even though it doesn't happen that much now, some people would stop taking medication and go to traditional healers instead.

Follow up question

Ingabe indzaba ye ukhandze kutsi yalaba labahlala emakhaya ikakhulu noma estsenjini nawcabanga iyayidlala I role, lokwekutsi ukhandze kumbe nendzaba yekutsi funa u disclose nawhlala ukhandza kutsi ekhaya kunebafati labang4 wena kumbe ungumfati number 2 fute ke utjele bonkhe labanye labantfu, noma utjele lobabe sowsaba kutsi utawtjela bonkhe labanye bantfu mawcabanga loku kuyayidlala yini indzima.

Response

I think living in big families does play a role. Sometimes wives are afraid to tell their husbands because they worry that if they tell them the husband will tell the other wives and everybody will blame her for bringing HIV to the family.

Q 10. Do you think women generally receive support from their community or family members who are aware of their HIV status? In what ways?

C 1

It depends, some gets support from their children or their husbands. There are still a few people who don't feel supported but most people support each other because almost everyone knows someone who takes ARVs. Even when they come to the clinic, because they're from the same areas, they see each other. Before the clinic used to service TB and HIV patients only so they'd meet at the clinic but now we see everybody that's why they don't carry their files when waiting in line anymore. The nurses carry the files, they only take their health cards to the counting station.

Q 11. Do people generally treat those taking ARVs badly in your community? Or speak poorly/gossip about them

C 1

They gossip about them. That this one is taking ARVs.

Q 12. Please describe any issues regarding stigma with ARVs in your community.

C 1

I don't think there have been issues of stigma in these communities. I think it's because in the beginning we used to go to go around with the clinic car testing people in their home and sometimes transporting them to the hospital. So they got used to seeing each other then. Before the clinic was well equipped, we used to take them to good shepherd for testing and treatment. Now we've started doing test and start.

Q 13. Are there programs designed to help support people taking ARVs in your community? What kind of support do these programs give

C 1

The only program we have is mothers to mothers

Q 14. What strategies do you use to track people who are defaulting on treatment?

C 1

Ok we are trying, every day we generate a list of patients who were supposed to come to pick up their meds. So when we register them, we put a check mark to indicate who has arrived. At the end of the day, if somebody doesn't show up, we call them. If calling is unsuccessful, we track them, first with the comm ART staff, and then with the vehicle. We have someone who drives around to track on those that have defaulted. But sometimes it's very difficult to reach them, especially those who haven't disclosed…they run away and hide from us. Sometimes this can lead to accidental disclosure, that's why some patients don't like home visits. Because if the patient didn't disclose to the whole family and then a health worker or the clinic car shows up for follow-

up, often times family members get surprised and start asking questions. So this becomes very complicated.

Q 15. In your opinion, what kind of policies/interventions should be implemented to help resolve the structural (the system) and social barriers to HAART adherence?

C 1

I think lack a food plays a significant role. The place is so dry, there are no rains so people can't grow crops. They rely on buying food from the store and they don't always have the money to do that. There are also no jobs which make things very difficult. It would be very helpful if the government could provide food support for the people taking ARVs. When it comes to disclosure, I think we have to have a policy that compels people to disclose their status to their sexual partners; otherwise we will never get rid of HIV.

END!!!

Q 1. Tell me about the women in your community? (Probe for: demographics, atmosphere, what you feel proud about, what are some challenges?)

G 1 & 2

Most of the people we see are young and older women, as well as the youth. They're mostly illiterate and unemployed. If they work, they usually work part-time in the community.

Q 2. What is the nature of your usual interaction with the women you serve?

G 1

Uhm mainly it's the nurse client relationship that we have, we try to bring it down to brother sister relationship and try to include some social based relationships if there's something like that.

Q 3. What are some of the issues you see among women on HAART?

G 1

People are less likely to adhere to medication if they haven't disclosed their status to their partners and families. Also those who haven't joined support groups are likely to default. Some women may take the ARVs in secret, hiding them from their husbands and this makes it difficult for them to take them consistently. Some have reported lack of money for transport yet they don't want to use our community ART program because they are afraid of being found out they have HIV. This is common among younger adults and adolescents.

Follow-up question

So hhay kuts kumbe lak community babese sebaya hletjwa noma sebayabandlululwa tintfo letinjalo?

G2 Response

I don't think there still discrimination in communities, especially now that we have support groups. Most people are aware of HIV now and we've been having more people coming out and sharing their status in public. The problem we have with discrimination now is among the health workers who tend to display a negative attitude towards people taking ARVs. They say negative things about some of the patients in public and use derogatory words e.g. "lababo ART". Some of them stop taking their medication because of poor treatment by the nurses.

G 2

Another issue is that in some clinics, ART people are made to que in a separate line which makes some people uncomfortable. Especially those who haven't disclosed their status, they don't want to be seen standing in line for HIV positive people. Another problem is alcohol use. People who drink are usually nonadherent because I think it's harder for them to remember taking their ARVs. They are also more likely to forget their clinic visits.

Q 4. What is your sense about the women's attitude/feelings towards HAART? How about their spouses/friends/family members?

G 1

For the ones we service in the community, adherence is really high because we often see people who are stable and don't need regular check-ups with the doctor. The ones in this program take their treatment seriously. However, we do have people who are not 100% adherent in this program. Some of them don't have money to go to the hospital for refills so we feel compelled to take them.

G 2

I think in general, the women take their medication seriously. But when it comes to family members, it depends. Some families are accepting and supportive but others not as much. Often times women are easy to initiate than males.

Follow-up question

Lendzaba yalo tjwala niytsini nine niyakhona yin kuykhulumisa?

G 1 Response

When it comes to alcohol use it's a very difficult situation, particularly because even the health workers themselves, the people you expect to counsel them against drinking and to lead by example, are drunkards themselves. We try to educate people about the effects of alcohol on the medication…we tell them to not drink alcohol because it makes the medication not work properly, but how can you expect them to take us seriously when some health workers are doing the same.

Health workers should lead by example. We often tell them to wait at least 2 hours before and after taking the meds but we know they don't do it…some take the meds with alcohol.

Q 5. What kind of comments or stories do you hear from the women about HAART in general?

G 1

I like it when they talk about how ARVs have changed their lives, and how they encourage other patients to adhere to their medication. They are say positive things about their medication, and they are very grateful of it. I'm so proud of them.

Q 6. What are some of the reasons women give for not taking their medication consistently?

Discussed above.

Q 7. Do you think women skip taking their ARVs if they do not have enough food to eat? Do they mention lack of food as one of the reasons for not taking their ARVs?

G 1

Yes, patients have been reporting lack of food as a reason they don't take their pills.

Q 8. Do you think travelling long distances to the clinic and lack of bus fare make it difficult for women to adhere to medication? Please elaborate. **G 1**

Long distance travel is not that big of a problem now that we do refills in the community but it was a big problem before we started the program. Even now, we still have people who are defaulting because of that.

Q 9. Do you think cultural traditions/societal norms (e.g. polygamy) play a role in whether or not women take HAART? If so, how?

I think cultural traditions play a role. Our culture allows men to have as many girlfriends and wives as they want and that contribute to the spread of the disease. Also having multiple sex partners may contribute to medication nonadherence especially for people who haven't disclosed their status. This is also true among women in polygamous marriages. Sometimes it's difficult for the women to disclose their status to their husbands let alone the other wives in fear of being accused of infecting everyone with HIV in the family (even though nobody knows who was infected first), so these women end up keeping their status to themselves and taking their meds in secret.

G 2

To add on the cultural traditions, some people still believe in traditional medicine. They tend to either not take the ARVs because they believe the traditional concoctions will cure them or they mix the meds with these concoctions. Either ways this affects adherence.

Q 10. Do you think women generally receive support from their community or family members who are aware of their HIV status? In what ways?

G 1

I think in this community it's a big yes. The people here are very supportive of each other. The building that you guys used for the interviews the community gave it out to support such activities. I think they're as supportive as they can afford.

Q 11. Do people generally treat those taking ARVs badly in your community? Or speak poorly/gossip about them?

G 2

Yes people gossip about them, they say all kinds of negative things about people taking ARVS. They even have derogatory terms for it like "phinduvuke, hohlohohlo etc". I think it's more in town than in the rural areas though.

Q 12. Please describe any issues regarding stigma with ARVs in your community.

G 1

I think it's the issue with the bad health workers behavior has a big influence, followed by the self-perceived stigma. The self-perceived stigma is so high such that people are even ashamed of being seen anywhere near a hospital vehicle, and they don't want the hospital car to visit them at their homes. Like the car we're using for comm ART, it's not written good shepherd outside because of that reason.

Q 13. Are there programs designed to help support people taking ARVs in your community? What kind of support do these programs give?

Except for the RHMs, nothing that we know of. People just try supporting each other whenever they can but it's hard. With such high unemployment rates, there's no money and everybody's poor.

Q 14. What strategies do you use to track people who are defaulting on treatment?

G 1

Because we're short staffed, we have defaulter tracing officers who do the follow-ups. They use motorbikes to conduct home visits.

Q 15. In your opinion, what kind of policies/interventions should be implemented to help resolve the structural (the system) and social barriers to HAART adherence?

G 1 & 2

- Staff training to teach them about how they can better serve their clients
- Improve patient services by adopting a client cantered approach
- Accountability for health workers
- Laws to compel disclosure status among people living with HIV

END!!!

Q 1. Tell me about the women in your community? (Probe for: demographics, atmosphere, what you feel proud about, what are some challenges?)

M1

Most of them are uneducated. They don't know a lot of things; they don't even know their rights as people living with HIV. Some of them have never been to school, they don't have IDs and don't even know their age.

Follow-up question

Uma utsi abati emalungelo abo usho kuts, I example nje?

Response

For example, it seems like they don't know that its their right to demand protection. If those living with their partners ask to use a condom and the husband says no, they don't stand up for themselves. Yet they know they need to use a condom to prevent the spread of the disease. Not just HIV but STIs as well. They think they have to do as their partners say since they're married to or living together this person. Sometimes a woman comes in, they have an STI and the nurses tell her not to have sex for a certain period of time, you find that the husband refuses. There is a lot going on, it just seems like these women are not empowered.

Follow-up question

Uma ubuka khona yin ema challenges ukhandze kuts kumbe umangabe umuntfu afikile nimcale ku medication ukhandze kuts afike atsi lobabe cha angiyfuni kemine lentfo leyo, niya hlangabetana yin nema challenges lakanjalo? Khandze kuts soyambona senimcalile atsi sonetinkinga tekbuya ngalokuts nawtsi wena ngazuzts abakhoni loktimela kuyak'affecta yin lok indlela labatsatsa ngako lama arvs? Ngekbuka kwakho as a health worker.

Response

Because these women are not empowered, it also affects adherence to treatment. Sometimes we start someone on HIV medication and they don't tell their partners. They take the pills secretly but it's difficult because they have to hide when taking them. They have to wait for the right opportunity and sometimes they don't get it so they skip taking the medication.

Q 2. What are some of the issues you see among women on HAART?

M 1

They often say they don't have money for transport to come to the clinic. Sometimes they say they were away visiting relatives and they forgot their appointment dates. But they often say it's the lack of money. Sometimes they their bosses at work don't allow them to come to the clinic to get their medication because they don't care whether or not someone is taking ARVs they just want the job done.

Follow-up question

Emakhaya ke khona yini labaye bak'khulumise in terms kutsi ekhaya khona kute tinkinga labaye batisho?

M2 Response

Most times they complain about lack of food, they say they can't take the meds when they don't have anything to eat. But another big problem here at Meni are the firms, their bosses don't want them to take a day off to come pick-up their meds every month, even if we give them a sick note.

Q 3. What is your sense about the women's attitude/feelings towards HAART? How about their spouses/friends/family members?

M 1

They appreciate them a lot. They even compare their weight to see how they're doing from when they started until now. They appreciate them a lot, they understand it's a life and death situation. With regard to that, there's not a problem.

Q 4. What kind of comments or stories do you hear from the women about HAART in general?

M2

Nothing except that some people still have stigma of being found out that they're taking ARVs. They don't want to be seen carrying the pills, they say people can hear them because the pills make noise. They often take them out of the container and put them in the regular plastic packets.

Follow-up question

So nanbuka nine nje as ema professionals health professionals, sisekhona yin stigma vele lak'community, do they get stigmatized or discriminated just because badle maphilisi? Yini leleyenta kuts besabe lokuts bantfu beve aba hohlohohlo maphilis mabawa phetse?

M1 Response

I don't think there is stigma in the community. I think the patients have self-perceived stigma.

Q 5. What are some of the reasons women give for not taking their medication consistently?

M 1

They don't say anything, they say they take them even though the records show that they have not been adherent. I often encourage them to tell me the truth so I can help them and they say they sometimes forget taking them, sometimes even for days.

Follow-up question

Bavama nje nglokuts bayakhohlwa bats bayawa khohlwa?

Response

In some cases, even if they don't forget taking their meds, some report lack of food. They say they can't take the pills if they haven't eaten.

Q 6. Do you think travelling long distances to the clinic and lack of bus fare make it difficult for women to adhere to medication? Please elaborate.

M 1

Yes, lack of money for transport makes them skip clinic visits. Some live in areas where there's no public transport they walk long distances through mountains to get to the clinic. Some of them so if they're feeling sick that day, they don't show up to the clinic. Some live in areas where there's no public transport at all so they have to walk.

Q7. Do you think women skip taking their ARVs if they do not have enough food to eat? Do they mention lack of food as one of the reasons for not taking their ARVs?

Yes, addressed in Q5.

Q 8. Do you think cultural traditions/societal norms (e.g. polygamy) play a role in whether or not women take HAART? If so, how

M 1

I'm not sure but sometimes it happens with the ones who are newly initiated. They get started on ART and sometimes put it aside and go to see traditional healers. Then once they get sick again, they come back to the hospital.

Q 10. Do you think women generally receive support from their community or family members who are aware of their HIV status? In what ways?

M 1

They say they have support groups where they meet and talk about their problems but I'm not sure where they meet.

M 2

I haven't seen anything; I don't think there's stigma in the communities. I think people have accepted that ARVs are a part of life. They understand that they are just like taking any other medication, just like taking diabetes or hypertension meds. They understand that when you adhere to the ARVs you'll get healthy and live a long life. They know they're as important as other meds.

Q 12. Please describe any issues regarding stigma with ARVs in your community.

M 1

I think if it were possible to have mobile clinics for remote areas that would be great. That way it people living far away can get their refills without worrying about money.

Q13. Are there programs designed to help support people taking ARVs in your community? What kind of support do these programs give?

M2

None.

Q 14. What strategies do you use to track people who are defaulting on treatment?

M1

We call via their cell phones and conduct home visits. We have community health workers who do close follow-ups on them.

END!!!

Q1. Tell me about the women in your community? (Probe for: demographics, atmosphere, what you feel proud about, what are some challenges?)

S1

OK our clients are different some show negative attitudes towards us. By having an attitude I mean she always show up late to the clinic because she never disclosed her status. So when she comes to the facility she has to come up with an excuse like telling her partner she's going to the shops. So maybe they show up say ten minutes to lunch time, and sometimes even the health worker and that makes the health worker to have a negative attitude towards that client. But women who disclosed, are free, their adherence is 100% they just they don't have a problem.

S 2

I just want to add on the problem of disclosure. I think disclosure is a major challenge because its very difficult for the client to adhere to the treatment if they haven't told anyone about families status. Another thing is that some of the clients drink alcohol and you find that they do not adhere to appointment visits because of the drinking, they forget the dates. Sometimes, I think past experiences also play a role. Especially with the pmtct, some clients that have negative children and were positive when they had those children and didn't take the meds yet the children were

born negative. Sometimes when doing the pill count, the numbers are off. They tell me they didn't put the meds aside/ throw them away so when they say that I know they need counselling.

Q 2. What is the nature of your usual interaction with the women you serve?

S 1

I see HIV positive pregnant and lactating mothers. They see us as part of their prenatal care. We do HIV counselling, testing, and provide treatment refills. We teach them about mother to child prevention and HIV treatment.

S 2

I share our past experiences with them as a person living with HIV. And I also encourage them that it's better to start taking treatment as soon as possible, before they get too sick. I think sharing our past experiences with them helps them a lot. They feel comfortable when we talk, like they hear it from the horse's mouth.

Q 3. What are some of the issues you see among women on HAART?

S 1

I think it goes back to the disclosure issues as well as self-perceived stigma. Some people don't want to be found out they have HIV. They don't want to be seen here at the clinic standing in line to get ART because some of them still haven't disclosed to their families. Also poor service at the clinic might be driving some people away. When they come here looking for help and they're not treated well by the staff, they go back and don't come back, sometimes for months on end. Sometimes they get yelled at when they arrive at the clinic late. Yet there are things that make them get to the clinic late, things like the issues of disclosure and long distance travel.

Q 4. What is your sense about the women's attitude/feelings towards HAART? How about their spouses/friends/family members?

S 1

It goes back to the issues of disclosure. You find that the women are taking the meds in secret because they haven't disclosed their status to their partners or families. They know it is important that they take treatment and importance of disclosing their status so we counsel them and encourage them to join support groups so that they know they are not alone. We also encourage them to bring their partners and families to the support group as well.

S 2

And sometimes you find that there is accidental disclosure. Sometimes the clients lie about disclosure. When we fill in the treatment supporter information, we ask the patients to give us a name of someone they trust and have disclosed their status to so that when the patient stops coming to the clinic we can call them for a follow-up. And you find that when we call the number they gave us, the person on the phone either doesn't know the client or they are not aware that they've been registered as the patient's treatment supporter. Sometimes it's very difficult to reach

them, especially those who haven't disclosed…they run away and hide from us… they don't like home visits. Because if the patient hasn't disclosed to the whole family and then a health worker or the clinic car shows up to do a follow-up, often times family members get surprised and start asking questions. So this becomes very complicated.

Follow up question

Laba lenbabutako mangabe banbhobhokele bayaye batsi yini tinkinga letenta kutsi bangabuyi l clinic ngaphandle kwe disclosure, khona yini letinye nje?

Response

Other problems are lack of money and lack food. Most people say they stopped taking ARVs because they didn't have money to buy food. They complain that the medication make them sick if they take it hungry. That's why we avoid initiating people who live far away because they tend to default in treatment because they often don't have money for transport.

Response

Another thing is medication side effects. Some of them hear people talking about the negative effects of the medication, like people say the meds makes tummies big and gives them humps at the back, so they get scared and stop taking the pills. Some people complain that the medication gives them deformities in different parts of the body (face, back, hips, legs, etc) so they don't like it.

Q 5. What kind of comments or stories do you hear from the women about HAART in general?

S 2

Also alcohol use plays a role. Alcohol makes them forget their clinic appointments. I also think that it also makes them forget taking their medication on time at home.

Question 6 and 7 skipped because they've already been answered.

Q 8. Do you think travelling long distances to the clinic and lack of bus fare make it difficult for women to adhere to medication? Please elaborate.

S 1

Yes they report lack of money for bus fare as one of the reasons they miss their clinic visits.

Q 9. Do you think cultural traditions/societal norms (e.g. polygamy) play a role in whether or not women take HAART? If so, how?

S 1

Yes I think cultural traditions play a role but not that much. People have been educated about not mixing traditional concoctions with ARVs. When waiting on the benches outside, you hear them talking among themselves saying that it's not good to mix the traditional medicine with the ARVs.

Q 10. Do you think women generally receive support from their community or family members who are aware of their HIV status? In what ways?

S 1

I think they do get support from partners and family members because when we initiate them to treatment we ask them to give us a person we can put on file as their treatment supporter. We tell them to give us a name of someone they trust and they can count on for support.

Q 11. Do people generally treat those taking ARVs badly in your community? Or speak poorly/gossip about them?

S 1

I think there has been a significant decrease with regard to stigma or discrimination in the community. When we ask the patients if they feel they get discriminated against they say no. Even though people may not be discriminated against in the community, most people are self-discriminating/ stigmatizing.

Q 12. Please describe any issues regarding stigma with ARVs in your community.

S 1

There seem to be no stigma in communities anymore. Most people are aware of HIV and know someone living with HIV. Some people are not ashamed to talk about their status even among people they don't know, for instance when they are standing in line at the clinic you hear them talking to their friends that they are at the clinic to pick up their medication.

Q 13. Are there programs designed to help support people taking ARVs in your community? What kind of support do these programs give?

S 1

Yes, we have community expert clients who are hired by the clinic to provide support to people living with HIV at the community level.

Q 14. What strategies do you use to track people who are defaulting on treatment?

S 1

Yes, we keep an appointment register which tells us who is supposed to show up at the clinic that day. If a patient doesn't show up we call them and make a follow-up. We also make follow-ups using the expert clients.

Q 15. In your opinion, what kind of policies/interventions should be implemented to help resolve the structural (the system) and social barriers to HAART adherence?

S 1 & 2

- **Counselling**

- **Educate people about the importance of ARVs**
- **We need to implement strategies reduce waiting time at clinic. Like today, everybody is at a meeting and I'm all by myself. I can't keep up with the clients.**
- **Also the clinic needs more workers. Some clients complain about the waiting at the clinic. We are understaffed here so sometimes it takes a long time to help everybody.**
- **Continue with community ART**

END!!!
